# Monthly Summary

**Published:** 1896-06

**Authors:** 


### Monthly Summary.

### HYDROZONE IN PURULENT OTITIS MEDIA.

### A REPORT OF A CASE SUPPOSED TO INVOLVE INFLAMMATION OF THE MASTOID.

BY WM. CLARENCE BOTELER, M. D., KANSAS CITY, MO.

On November 4th, 1895, I was consulted at my office by Robert P——, aged 24 years; occupation, laborer in the Armour Packing Company. The patient complained that for about four weeks he had been suffering from intense pain in his left ear, making it impossible for him to sleep at night, or rest during the day. The pain was so severe that at times he apparently lost consciousness and it seemed to extend through his entire brain. Upon inspection, the man's face was found terribly deformed; an edematous the size of one-half of an ordinary loaf of baker's bread occupied the usual location of the ear and the surrounding muscles. The auricle of the ear was almost buried in edematous tissue; upon palpation, the part was found intensely tender, and deep pressure evoked expressions of excruciating pain. The integument and sub-cutaneous tissue were thoroughly infiltrated. Ichorous, fetid pus was slowly exuding from an almost imperceptible meatus. The patient expressed feelings of chilliness, showing a possible septic contamination of his system. Every indication and sign pointed to possible suppuration of the mastoid cells—tenderness upon pressure over the mastoid being very marked. Efforts to localize the tenderness, whether in external meatus or mastoid, for discriminating diagnosis, were unsatisfactory. I concluded to withhold a positive diagnosis as to whether the condition was purulent otitis media or suppurative imflammation of the mastoid, and used tentative treatment for a short while. I immediately placed the patient under heroic doses of elixir of the six iodides internally. After laborious effort I succeeded in separating the edematous tissue sufficient to admit the introduction of a small Eustachian catheter into the external meatus. Through this with a small hard rubber syringe, I injected four times daily about one-half of an ounce of hydrozone, allowing it later to drain away, advising hot fomentations. The patient was confined to his bed and the best possible hygienic surroundings provided. In twenty-four hours after the treatment was commenced, the intensity of the odor, amount and character of the discharge had manifestly lessened, the swelling was reducing and the patient feeling better. The edema being lessened, the aperture was enlarged. I now recommended the injection of hydrozone through the catheter of larger calibre, every hour, requiring the head to be turned to the opposite side for ten minutes to allow the percolation of the hydrozone as deeply as possible into the middle ear, before reversing the position to allow drainage. We continued this treatment for a week, the man's recovery progressing with remarkable rapidity, his pain and the constitutional symptoms having disappeared about the third day. At the end of eight days the swelling had entirely disappeared, his features were again normal, and he expressed himself as perfectly well. An examination showed a circular perforation in the ear drum the size of a shot, proving that the cause had been one of purulent otitis media, with septic contamination of the patient's system, and infiltration of the surrounding cutaneous tissues. Small incisions were made at two different places to permit the exit of pus from the integument. The mastoid was found not involved. The rapidity with which the disease yielded after the introduction of hydrozone through the catheter into the middle ear impressed me with the wonderful value of the preparation; for, struggling with such a case during a practice of seventeen years, I have never seen its efficiency equalled by any medicinal or operative procedures.—Published by the *Medical Bulletin* of Philadelphia, Pa., February, 1896.

### PEROXIDE OF HYDROGEN.

BY J. P. PARKER, PH. G., M. D., OF ST. LOUIS, MO.

Published by the Annals of Ophthalmology and Otology, of St. Louis, Mo., April, 1895.

(Abstract from *The Times and Register*, June 8th, 1895.)

I have used peroxide of hydrogen quite extensively for cleansing discharging ears, the nasal and accessory cavities, and have tried all the brands of the preparation in the market, and once thought one manufacturer's make as good as that of another, and bought the cheapest as a matter of economy, but recent experience has taught me that the difference in quality is greater than the difference in price. After an unpleasant experience with a solution of peroxide of hydrogen which severely injured the mucous membrane, I bought and examined, chemically, a bottle of each preparation of $H_2O_2$ in the market, and was surprised to find so much difference. Some are useless, and others worse than useless because they contain too little available oxygen and too much free acids (phosphoric, sulphuric, hydrochloric). I now order Marchand's (medicinal) exclusively because I find it contains the desired quantity of available oxygen and not enough free acid, to be objectionable, and its keeping properties are all that could be desired.

By inquiry I learn that Marchand's is the preparation that is used by almost all surgeons, and it is considered by them the standard.

—[My personal experience with peroxide of hydrogen confirms entirely the statement of Dr. J. P. Parker, I have used exclusively Marchand's brand until lately, when I experimented with hydrozone. Then I gave up entirely the use of peroxide of hydrogen and use hydrozone on account of its strength, which cannot be compared with any other brand, even Marchand's. I must say that the results which I obtained with hydrozone are most gratifying.—ED].

